# World Health Organization's Early AI-supported Response with Social Listening Platform

**DOI:** 10.5195/jmla.2022.1398

**Published:** 2022-04-01

**Authors:** Bethany S. McGowan

**Affiliations:** 1 bmcgowa@purdue.edu, Associate Professor, Libraries and School of Information Studies, Purdue University, West Lafayette, IN

## Abstract

**World Health Organization's Early AI-supported Response with Social Listening Platform** (WHO EARS). WHO HQ, Avenue Appia 20, 1211, Geneva 27, Switzerland; https://www.who-ears.com/; free.

## PURPOSE

As trusted community partners and expert information providers, listening and responding to community information needs is a core tenet of librarianship. Social listening is the process of extracting information from social media channels to inform the real-time measure of social developments via the detection of emotions, topics, and opinions; the mapping of information flow; and/or the modeling of opinion networks [1]. Librarians have used social listening to inform the design of adaptive services, plan programs, and improve community engagement. For example, in her presentation at the 2016 Library Marketing and Communications Conference, Marketing and Student Outreach Librarian Maria Atilano discussed how using tools like TweetDeck, Feedly, and Hootsuite for social listening allowed her to hear what was being said about her library. Social listening allowed her to gather the information needed to understand student needs and foster a sense of community [2]. Relatedly, a 2020 webinar from ALA's Public Library Association highlights social listening dashboards, with their automation of insight gathering from across a range of social media platforms, as an important provider of qualitative and quantitative elements that can help librarians develop relevant services [3]. The World Health Organization Early AI-supported Response with Social Listening Platform (WHO EARS) is a social listening dashboard for health information professionals that can inform the design of timely and engaging health literacy interventions by highlighting real-time COVID-19 narratives happening in public spaces online.

## GENERAL DESCRIPTION

The WHO EARS platform allows users to explore conversations in public spaces online at a global or country level. Each day, data from COVID-19 conversations are automatically collected from sources like blogs, news comments, online forums, and public social media posts. The EARS platform allows users to choose between a country-specific dashboard for detailed analysis or an open API dashboard with aggregated, anonymized data for a broader picture. These dashboards

show how the topics of conversation change and evolve country by country over time, such as: what are the most popular categories and those gaining traction, and their patterns; what are the top and rising terms and hashtags within each category; what are the differences in conversations by; the composition of the conversation by intention: questions (confusion), complaints (frustration) or praise [4].

When EARS launched in January 2021, it included data from twenty WHO member nations, collected in English, Spanish, French, and Portuguese. Data from other countries and languages are being added, and as of September 2021, the platform covered thirty countries.

Each country sample includes approximately 3,000 opinions per 1 million inhabitants per month, with exceptions for small countries for which the sampling ratio is higher. Data are automatically categorized into forty categories using a semisupervised machine learning algorithm. The categorization includes human quality controls and is based on a public health social listening taxonomy. Additional details related to data sources, processing, aggregation, and presentation are available on the EARS platform.

## INTENDED AUDIENCE

The WHO EARS platform is designed for multidisciplinary use by health information professionals, providing real-time information of COVID-19 narratives “so we can better manage as the infodemic and pandemic evolve” [5]. The World Health Organization defines an infodemic as “too much information including false or misleading information in digital and physical environments during a disease outbreak” [6]. Infodemics can lead to negative health outcomes and hamper public health response by causing confusion, increasing risk-taking behavior, and increasing mistrust in health authorities. These effects can be worse in marginalized and vulnerable communities [7]. Related, infodemic management is “the systematic use of risk- and evidence-based analysis and approaches to manage the infodemic and reduce its impact on health behaviours during health emergencies” [8].

The EARS platform allows librarians to support infodemic management by providing real-time detection of emotions, topics, and opinions. The platform includes a breakdown that highlights COVID-19-related categories of conversation, how trends for each have changed over time, and top keywords, rising keywords, top hashtags, and rising hashtags for each category in a specific country or world region. With a broad idea of public sentiment, librarians are better equipped to provide timely, efficient services that answer popular questions.

## LIMITATIONS

Although EARS is global and country focused, coverage is currently limited to thirty countries and eight languages. It does not include state, regional, or community-level information.

As with other social listening dashboards, there are ethical considerations, especially as related to transparency and privacy. And, because dashboards condense complex data into a simplified format, they risk skewing the accuracy of interpretation [9].

## TECHNICAL REQUIREMENTS

The WHO EARS platform is browser-based and does not require any downloads or logins. The website is mobile-friendly—optimized for smartphones, tablets, and similar handheld devices. Features include downloadable datasets, which require the use of spreadsheet software. EARS encourages the use of Facebook or Twitter to share social listening insights, and users may find that accounts with one or both of those social media tools are useful, though not necessary.

## MAJOR FEATURES

**Figure 1:**
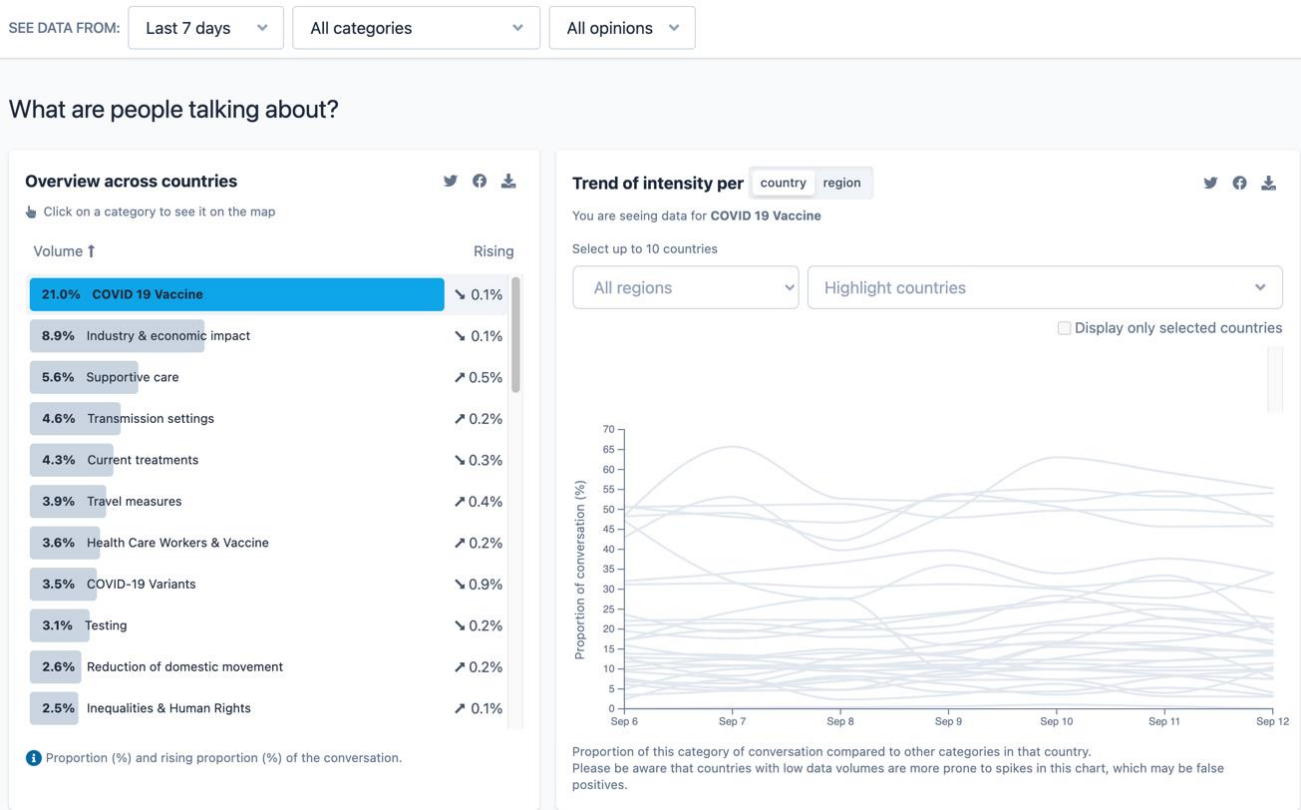
EARS graphs illustrating COVID-19 topics being discussed across 30 countries

Figure 1 is a screenshot of a portion of the EARS toolkit, illustrating what people are discussing in the last seven days, across all categories and opinions. Major features of the EARS platform include the following:

1. Options to choose from a global overview or country report.
2. Options to sort data by
  - date range, with options for seven days or thirty days.
  - category, with options for cause, illness, treatment, intervention, or type of information.
  - opinions, with options for questions or complaints.
3. What are people talking about?
  - Includes data from thirty countries (as of September 2021) and seven regions—Africa, Americas, Eastern Mediterranean, Europe, South-East Asia, and Western Pacific.
  - Includes an analysis that lists forty categories, sorted by volume (with highest volume first), and reports if the category is rising or falling in popularity.
  - Reports the trend of intensity for each category—the proportion of conversation per day—in each country or region.
4. What are the differences?
  - Compares how interest in topics vary across countries.
  - Can be compared according to volume, rising trends, or gender gap.
5. Other interesting features include links to access the data for custom analysis via API or GitHub; options to suggest data for inclusion; the opportunity to provide feedback about the project; more about the data, limitations, and how to interpret it; and the option to subscribe to the WHO Infodemic Management newsletter.
